# Circular RNA hsa_circ_0002268 (*PHACTR1*) Is Specific to Gestational Diabetes Mellitus in a Polish Pregnant Population

**DOI:** 10.3390/ijms25137040

**Published:** 2024-06-27

**Authors:** Dominik Franciszek Dłuski, Marek Cieśla, Dorota Darmochwał-Kolarz

**Affiliations:** 1 Department of Obstetrics and Perinatology, Medical University of Lublin, 20-090 Lublin, Poland; 2 Institute of Medical Science, College of Medical Science, University of Rzeszow, 35-959 Rzeszow, Poland; mciesla@ur.edu.pl; 3 Department of Obstetrics and Gynecology, College of Medical Science, University of Rzeszow, 35-301 Rzeszow, Poland; ddarmochwal@ur.edu.pl

**Keywords:** GDM, pregnancy, circRNA, PHACTR1, hsa_circ_0002268

## Abstract

Gestational diabetes mellitus (GDM) is an intolerance of carbohydrate of any degree, which appears for the first time or is diagnosed during pregnancy. The objective of this study is to assess the differences in circular RNA (circRNA) in a Polish pregnant population with and without GDM. A total of 62 pregnant women, 34 with GDM and 28 controls, were enrolled in the study. Total RNAs were extracted from plasma and reverse transcription to complementary DNA (cDNA) was performed. A panel covering 271 amplicons, targeting both linear and circular as well as negative control gene transcripts, was used. Next-generation sequencing was used to evaluate the circRNA quantity. Data analysis was performed using the Coverage Analysis plugin in the Torrent Suite Software (Torrent Suite 5.12.3). A two-step normalization was performed by dividing each transcript read count by the total number of reads generated for the sample, followed by dividing the quantity of each transcript by β-actin gene expression. Both circular and linear forms of RNAs were independently evaluated. A total of 57 transcripts were dysregulated between pregnant women with GDM and controls. Most of the targets (n = 25) were downregulated (cut-off ratio below 0.5), and one target showed a trend toward strong upregulation (ratio 1.45). A total of 39 targets were positively correlated with fasting plasma glucose (FPG), but none of the tested targets were correlated with insulin, CRP or HOMA-IR levels. Among the pregnant women with gestational diabetes, the relative quantity of hsa_circ_0002268 (*PHACTR1*) was approximately 120% higher than among healthy pregnant women: 0.046 [0.022–0.096] vs. 0.021 [0.007–0.047], respectively, (*p* = 0.0029). Elevated levels of hsa_circ_0002268 *(PHACTR1)* might be specific to the Polish population of pregnant women with GDM, making it useful as a potential molecular biomarker in the management of GDM in Poland.

## 1. Introduction

Gestational diabetes mellitus (GDM) is an intolerance of carbohydrate of any degree, which appears for the first time or is diagnosed during pregnancy. O’Sullivan was the first person who used this term, in 1961 [1]. The global GDM prevalence in the world population is estimated at 14.0% [2], but it depends on the country (from 2.2% in Sweden to 49.5% in Saudi Arabia) [3].

GDM is related to pregnancy complications such as prematurity, preeclampsia, hypertension, birth trauma, shoulder dystocia and fetal macrosomia [4,5]. Additionally, GDM is related to a higher probability of a C-section, and greater risk of prenatal and perinatal mortality [5]. Neonate complications such as a respiratory distress syndrome (RDS), hypoxia, or hypoglycemia are also connected with GDM [6]. 

The negative influence of GDM can be detected in the future life of the child and the mother. Patients with GDM have a higher risk of metabolic syndrome development, or type 2 diabetes mellitus (T2DM) [7,8]. Scientists estimated that up to 70% of women with GDM will develop diabetes mellitus within 22–28 years after pregnancy [9,10,11,12]. The prevalence of GDM increases with risk factors such as sedentary lifestyles (low physical activity, inadequate diet), obesity, emerging environmental factors, and the increasing reproductive age of women [9,10,11,12,13,14,15]. Similar problems, including obesity, metabolic disease, T2DM, and cardiovascular disease (CVD), have been observed in children born to women with GDM during their maturity [5,16,17]. There is also increasing evidence connecting GDM with abnormal brain development, with consequences such as concentration problems [18] or effects on general cognition [19]. 

The International Association of Diabetes and Pregnancy Study Groups (IADPSG) created one of the most common and widely used set of diagnostic criteria for GDM. According to these recommendations, an oral glucose tolerance test (OGTT) with 75 g glucose is performed at 24–28 weeks of gestation in every pregnant patient. Three plasma glucose values are assessed: fasting, 1 h, and 2 h post glucose. If any one of them is abnormal (fasting glucose, mmol/L (mg/dL) ≥ 5.1 (92); 1 h glucose, mmol/L (mg/dL) ≥ 10 (180); 2 h glucose, mmol/L (mg/dL) ≥ 8.5 (153)), we can diagnose GDM [20]. The existence of a strong correlation between maternal hyperglycemia and complications of pregnancy, which was analyzed in clinical studies, helps to individualize the cut-off values for the GDM diagnosis [5].

Circular RNA (circRNA), a type of noncoding RNA, is a closed cyclic structure in which a lack of 5′ and 3′ polar ends is observed. It has been widely observed in many organisms, especially in pants, nematodes, mice and humans [21]. CircRNAs may regulate gene expression levels by interacting with RNA binding proteins or by acting as a microRNA sponge. They are common in many types of tissues, for example in blood, serum, peripheral blood mononuclear cells (PBMC), exosomes, and other body fluids. Therefore, they are interesting molecules with the potential to be biomarkers that can support the diagnosis of various diseases [22].

CircRNA has attracted the interest of scientists as an important regulator of gene expression in many diseases, including cancer, atherosclerosis, and diabetes [23].

This research focuses on assessing the circular RNA patterns and their differences among pregnant women with and without GDM. We hope that the results can help to find a solution for better management of GDM.

## 2. Results

A total of 57 transcripts were dysregulated between the pregnant women with GDM and controls. The median expression ratios were calculated (median transcript expression in GDM patients divided by median transcript expression in controls) to visualize dependencies between the groups. Most of the targets (n = 25) were downregulated (cut-off ratio below 0.5), and one target showed a trend towards strong upregulation (ratio 1.45). The top 10 dysregulated transcripts are presented in Table 1. 

All transcripts for which the concentration was significantly different between groups are presented in Supplementary data Table S1. Logistic regression analysis showed that 3 (NA_PTN_2.4369, NA_PHACTR1_2.43094 and NA_KITLG_2.26281) out of 57 transcripts that were dysregulated could be used as potential markers of GDM (Table 2).

Due to the fact that both linear and circular forms for the gene *PHACTR1* showed dysregulation, the relative expression of the circular form was calculated based on the following equation: circRNA relative expression = circRNA concentration/(linear form concentration + circular form concentration). Among the pregnant women with gestational diabetes, the relative *PHACTR1* levels were approximately two-fold higher compared to the healthy pregnant women: 0.046 [0.022–0.096] vs. 0.021 [0.007–0.047], respectively, (*p* = 0.0029). 

**Figure 1:**
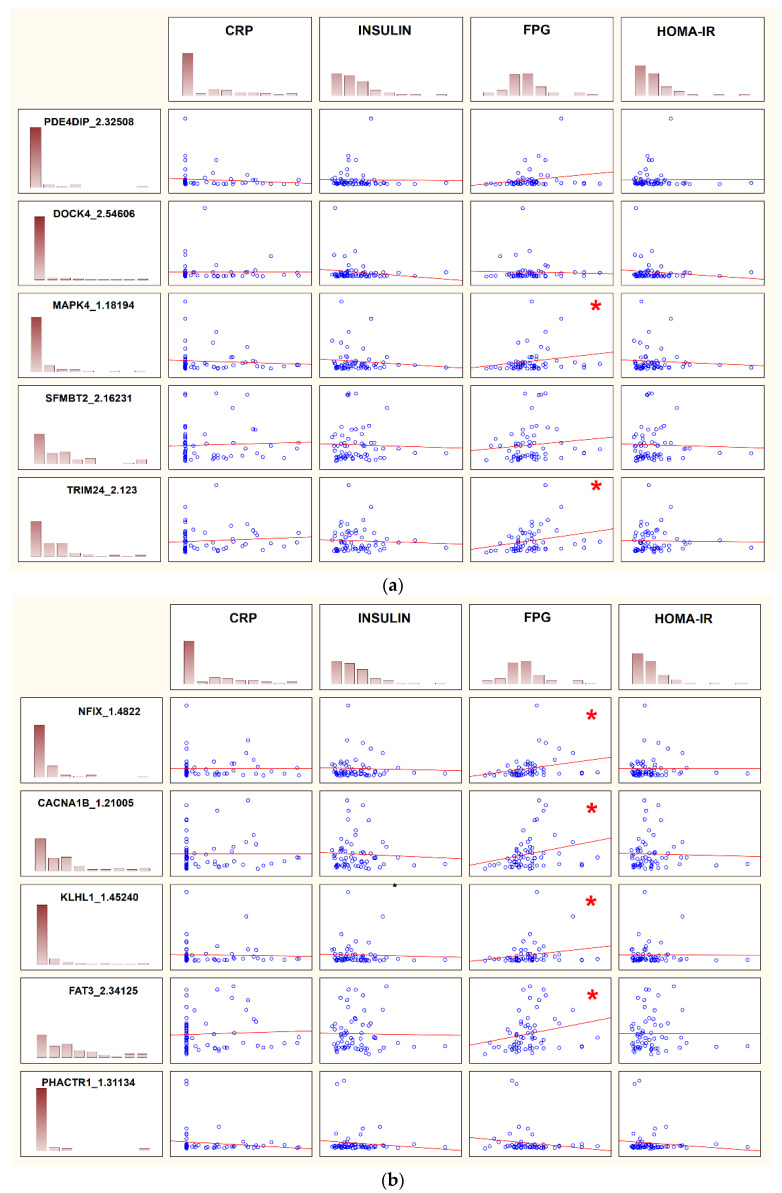
(**a**) Correlation between top 10 dysregulated transcripts and clinical variables (part 1). Abbreviations: CRP, C-reactive protein; FPG, fasting plasma glucose; HOMA-IR, Homeostatic Model Assessment of Insulin Resistance. Significant correlations are marked with a red star. Details are presented in Table 3. (**b**) Correlation between top 10 dysregulated transcripts and clinical variables (part 2). Abbreviations: please refer to Figure 1a. Significant correlations are marked with a red star. Details are presented in Table 3.

A total of 39 targets were positively correlated with FPG, but none of the tested targets were correlated with insulin, C-reactive protein (CRP) or Homeostatic Model Assessment of Insulin Resistance (HOMA-IR) levels (Supplementary data Table S2). Considering only the 10 most dysregulated transcripts from Table 1, only 6 of them showed a positive correlation with FPG. The correlations are graphically presented in Figure 1a,b. Detailed data are presented in Table 3.

However, no correlation was found between the selected transcripts from logistic regression analysis (PTN_2.4369, PHACTR1_2.43094 and KITLG_2.26281) or with the relative *PHACTR1* levels and clinical variables. Detailed data are included in Table 4. 

The expression levels of above-mentioned selected transcripts were evaluated between GDM treated by insulin (n = 20) and GDM treated by diet (n = 14). The analysis showed that the linear form of KITLG was upregulated in pregnant women with GDM compared to pregnant women without diabetes (median, [IQR]; 1.75 [1.17–2.39] vs. 0.76 [0.58–1.39], *p* = 0.02. However, both PTN and the relative level of *PHACTR1* showed a trend towards to dysregulation between groups. Details are presented in Table 5. 

Using the do prediction tool (available at: https://circinteractome.nia.nih.gov; access date 12 February 2024) [24,25], the set of 19 micro-RNAs (miRs) potentially interacting with phosphatase and actin regulator protein-1 (*PHACTR1*; circ RNA ID: hsa_circ_0002268, according to the Circinteractome database) has been identified. The detailed information is presented in Table 6. 

## 3. Discussion

### 3.1. Principal Findings

Among Polish pregnant patients with GDM, the relative quantity of hsa_circ_0002268 (*PHACTR1*) was elevated compared to controls. This indicates its possible involvement in the pathogenesis of GDM. 

### 3.2. In the Context of the Current Literature

CircRNAs may act as an miRNA sponge, protein-binding molecule, transcriptional regulator, protein scaffold, and protein function enhancer. Sometimes circRNA can be translated into functional protein [26,27]. Additionally, this type of RNA is profusely expressed in cells and tissues, and is relatively more stable than linear RNA, with a long half-life. CircRNA is specific to a given tissue, and is characterized by high evolutionary conservation among species, which suggests its usefulness as a biomarker in clinical applications [28].

Recent studies have reported that circRNA might play a crucial role in blood glucose level regulation, influencing insulin secretion and islet Beta- cell proliferation, which are pivotal in the pathogenesis of diabetes [29]. Xu et al. found that circRNA CIRS-7 has a strong influence on miR-7 inhibitor, and the Cdr 1 as/miR-7 pathway further favors production and secretion of insulin by regulating the insulin granule secretion target Myrip and boosting the insulin transcription target PAX6 [30]. Other researchers found that hsa_circ_0054633 was profusely overexpressed in the placentas and sera of GDM patients in the second and the third trimesters of gestation, and positively correlated with glycosylated hemoglobin levels and postprandial blood glucose levels [31].

Yan et al. evaluated the association between circRNAs and GDM pathogenesis using next-generation sequencing (NGS). They examined 48,270 circRNAs from placental villi and found that 255 circRNAs were downregulated and 227 were upregulated. The results showed the aberrant expression of circRNA in the villi of the placenta in GDM patients [23]. 

Jiang et al. performed microarray analysis to assess the expression of plasma exosomal circRNA 48 h before and 48 h after delivery. The hsa_circRNA 0039480 and hsa_circRNA0026497 were significantly expressed among GDM women before delivery. They also examined hsa_circRNA 0039480 and hsa_circRNA0026497 in different stages of gestation and found that hsa_circRNA 0039480 was highly expressed in every stage of pregnancy among GDM patients and positively correlated with OGTT results [32]. 

Bao et al. suggested in their study that circRNA DMNT1 may play an important role affecting trophoblast cells among patients with GDM and preeclampsia. They confirmed the up-regulation of circRNA DMNT1, which inhibited trophoblast cell proliferation, viability, invasion and migration, and promoted apoptosis [33].

Cao et al. analyzed the umbilical cord mesenchymal stromal cells (UmSCs) and circRNAs from exosomes among GDM patients. They found that the up-regulation of hsa_circ_0046060 reduced the intracellular glucose content in L-02 cells [34].

Wu et al., in their case-control study, assessed the expression level of hsa_circRNA_102682 in serum and checked its relation to lipid metabolism parameters. The hsa_circRNA_102682 was downregulated in GDM women and was significantly correlated with 1 h blood glucose level after OGTT, apolipoprotein A1 (apoA1), triglycerides, and apolipoprotein B (apoB). These results suggest that hsa_circRNA_102682 might regulate lipid metabolism and take part in GDM pathogenesis [35]. 

Huang et al. examined the association between levels of circSESN2 and IGF2BP2 and their effects on high glucose (HG)-treated trophoblast cells. Both of them were overexpressed among GDM women, which exacerbated HG-induced trophoblast cell damage [36].

Yang et al. analyzed the expression of hsa_circRNA1_102893, comparing GDM pregnant women with controls to assess this circ RNA as a diagnostic tool in early diagnosis of GDM. The areas under receiver operating characteristic (ROC) curves of hsa_circRNA_102893 were 0.806 and 0.741 in the training and test set, respectively, which showed that this circRNA has the potential to be a novel and stable noninvasive marker of GDM at early gestation [37].

Huang et al. assessed the exosomal circRNA circ_0074673 and its role in molecular mechanisms of GDM. They observed that exosomal concentration and size were greater in blood from the umbilical cord of GDM patients. The circ_0074673 expression was upregulated in GDM pregnant women exosomes and in human umbilical vein endothelial cells (HUVECs), which were co-cultured with exosomes. It was shown that the loss of exosomal circ_0074673 favored the angiogenesis, migration and proliferation of high glucose (HG)-HUVECs by regulating the miR-1200/MEOX2 axis. The results presented circ_0074673 as a new potential therapeutic target in patients with GDM [38]. 

Bao et al. suggested in their previous research that the circCHD2/miR-33b-3p/ULK1 axis may play a role in the pathogenesis of GDM [39]. In the next study, they investigated how it works. The observed that HG levels increased the expression of cicrCHD2, induced apoptosis and autophagy, and decreased viability in placental trophoblast HTR-8/SV neo cells. MiR-33b-3p was downregulated in GDM patient placentas by using HG and confirmed as a target for circCHD2 by qRT-PCR, a dual-luciferase reporter assay and bioinformatics analysis. UNC-51-like autophagy-activating kinase 1(ULK1) was identified as a target for miR-33b-3p using qRT-PCR, a dual-luciferase reporter assay, Western blot analysis and bioinformatics analysis. In contrast, ULK1 is upregulated in GDM patient placentas. The overexpression of ULK1 blocked the effects of mr-33b-3p mimics on cell apoptosis, viability, and autophagy in HG-treated HTR-8/SV neo cells. The data suggest that circCHD2 plays a role as an autophagy promoter via the miR-33b-3p/ULK1 axis in inducing apoptosis in HTR-8/SV neo cells, which presents cirCHD2 as a potential diagnostic and therapeutic target for GDM [40].

### 3.3. Potential Mechanisms

CircRNAs are important regulators of gene expression in many diseases including diabetes. Their dysregulation leads to changes in signaling pathways, glucose uptake, cell cycles and biological behavior in many human tissues. Hsa_circ_0002268 (*PHACTR1*) influenced protein phosphatase 1 catalytic subunit alpha (PPPCA1) activity, which is involved in the regulation in many cellular processes, e.g., glycogen metabolism [41,42] and cytoskeletal organization of pancreatic beta cells [43], which implies a role in GDM.

### 3.4. Implications

The frequency of GDM has been rising. OGTT is performed in the first trimester only in pregnant patients with the risk factors for GDM, while the standard time for testing is 24th–28th week of gestation. There are no reliable biomarkers which can be used to recognize GDM at the early time of pregnancy. The identification of phenotypes connected with GDM, described as the existence of risk factors, can be potentially helpful in modulating and customizing the diagnostic and therapeutic goals in pregnant women with GDM. We suggest that hsa_circ_0002268 might play this type of role. Both the circular form and relative expression of *PHACTR1* are independent indicators of other clinical parameters such as CRP, insulin, FPG, HOMA. Therefore, they can be considered as potential and additional markers of GDM.

### 3.5. Strengths and Limitations

The strength of this study is as a novel investigation of a Polish population (Caucasian race), whereas most previous studies were performed on Chinese populations (Han).

This study has several limitations: the small number of patients; patients were enrolled in one medical center; and the patients were of Caucasian race only. The results presented in this study should be validated in a larger number of patients. In addition, other methods should be considered, such as real-time quantitative PCR. In this study, the NGS technique was used as a screening tool to search for the most important transcripts, and this study should be considered as a basic study that will also serve as a reference point for other researchers. Further studies on an independent group of individuals are needed to confirm our results. The aim of this study was to determine the concentration of circRNAs in plasma as a material whose collection is not an invasive procedure for pregnant women. Moreover, the separation of the activities of cellular nucleases, mainly RNAses, makes the RNA material stable for a long period of time. On the other hand, it is necessary to examine other types of biological materials: peripheral blood mononuclear cells or umbilical cord blood. 

## 4. Materials and Methods

### 4.1. Sample Size Calculation

Circular RNA Research Panel: sequencing of circRNAs identified differences in gene expression between the two groups (patients with GDM vs. healthy pregnant women). Prior data indicated that the minimum average read counts among the prognostic genes in the control group was 5000×. The maximum dispersion was 0.3, and the ratio of the geometric mean of normalization factors was 1.

The Ion AmpliSeq Circular RNA Research Panel targets 271 amplicons related to key biological pathways. We assumed that the total number of genes for testing is 271 and that the top 27 genes (the minimum of 10% of overall amplicons) were prognostic. If the desired minimum fold change was 2.0, we needed 23 subjects in each group to be able to reject the null hypothesis that the population means of the two groups are equal with a probability (power) 0.8 by using the exact test. The false discovery rate (FDR) associated with this test of this null hypothesis was 0.01.

### 4.2. Patients

A total of 62 pregnant women, 34 with GDM and 28 controls, were enrolled in the study. GDM was diagnosed according to the IADPSG criteria with an oral glucose tolerance test (75g). The inclusion criteria for patients were as follows: singleton pregnancy, healthy patients (control group) or with GDM, no family history of diabetes, no fetal abnormalities, no comorbidities, and no infections. The exclusion criteria for the patients were as follows: infection, comorbidities diagnosed during gestation other than GDM, fetal abnormalities, family history of diabetes, and multiple pregnancy. The study was conducted in accordance with the Declaration of Helsinki, and the Ethics Committee of the Faculty of Medicine University of Rzeszow approved the study (protocol number 04/02/2020). The subjects provided written informed consent before any procedures. All of the individuals were from the local population (Caucasian race) from one research center. The subjects’ characteristics are presented in Table 7.

### 4.3. Assessment of Transcript Concentrations in Plasma

Whole blood samples with EDTA as an anticoagulant were collected and centrifuged at 3600 rpm for 10 minutes at room temperature to obtain the plasma. After that, the samples were stored at −80 °C until further analysis. Total RNAs were extracted using NucleoZOL reagent with NucleoSpin RNA Set (Macherey-Nagel, Düren, Germany) according to the manufacturer’s instruction, but the volume of 300 µl of plasma was used for extraction. At the beginning of each isolation, 1.5 µg of carrier RNA (polyadenylic acid, Eurx, Gdańsk, Poland) was added to each sample. Total RNA concentration was evaluated with the Qubit 4 instrument using the Qubit RNA Quantitation High Sensitivity Assay Kit (both from Thermo Fisher Scientific, Waltham, MA, USA). To evaluate the ability to amplify the selected targets, a quantitative polymerase chain reaction (qPCR) was performed using SG onTaq qPCR Master Mix (Eurx, Poland) in the QuantStudio 5 Real Time PCR System (Applied Biosystems, Waltham, MA, USA) under the thermal cycling conditions given in the mix manual. The GAPDH gene was used as a target. After the procedure, the RNAs were stored at −80 °C until downstream analysis.

### 4.4. Library Preparation

To perform reverse transcription of the total RNAs to a complementary DNA (cDNA), the Superscript VILO Kit (Thermo Fisher Scientific, Waltham, MA, USA) was used according to the manufacturer’s recommendation. After that, cDNA was used for library preparation. The equivalent of 15 ng of total RNA was used for the construction of the targeted library. This equivalent was prepared using the Ion AmpliSeq Circular RNA Research Panel (Thermo Fisher Scientific, Waltham, MA, USA), according to the manufacturer’s protocol. The panel, which covers 271 amplicons targeting both linear and circular as well as negative control gene transcripts, was used. The automatic library preparation was performed in the Ion Chef instrument with the DL8 library preparation kit. The samples were then pooled, which was followed by emulsion PCR using the Ion Chef System and Ion 540™ Kit-Chef, and sequenced on the Ion S5 System using Ion Torrent superconductor technology (all reagent and instruments were from Thermo Fisher Scientific, USA). Data analysis was performed using the Coverage Analysis plugin in the Torrent Suite Software. To ensure that the transcript quantity data were comparable between samples, a two-step normalization was performed by dividing each transcript read count by the total number of reads generated for the sample. This was followed by dividing the quantity of each transcript by β-Actin gene expression. Using this approach, both circular and linear forms of RNAs were independently evaluated. 

### 4.5. Statistical Analysis

Data distribution was assessed by the Shapiro–Wilk W test and quantitative variables with a normal distribution were presented as mean ± SD. Otherwise, the median (lower–upper quartile) was used. The differences between two independent groups were compared by Student’s *t*-test or the Mann–Whitney U test. Out of all transcripts, only 14 showed a distribution consistent with the normal distribution, but due to the fact that the studied groups were small and unequal, it was decided to use non-parametric tests for all the tested targets. The relationship between two continuous variables was analyzed by Spearman’s rank correlation, and only correlation coefficients (rs) that were significant are presented. Qualitative variables are given as numbers with percentages and were calculated using contingency tables with a χ2 test and Yates’s correction. Based on the obtained concentration results of the tested transcripts in the control group, a reference interval was calculated between the 5th and 95th percentile. On this basis, the positive (qualitative) expression of a given marker was determined and logistic regression was used to estimate the odds ratio (OR) and confidence interval (CI). P-values less than 0.05 were considered statistically significant. The analysis was performed with STATISTICA Version 13.1 (Dell Inc. 2016, Tulsa, OK, USA).

## 5. Conclusions

Elevated levels of hsa_circ_0002268 *(PHACTR1)* might be specific to GDM for a Polish pregnant population, making it useful as a potential molecular biomarker for the management of GDM in Poland, but further research is needed to confirm our findings.

## Figures and Tables

**Table 1:** The top 10 dysregulated targets between GDM and controls.

Target	GDM (n = 34)			Controls (n = 28)			Ratio GDM/Controls	*p*-Value GDM vs. Controls
	Median Concentration	Lower Quartile	Upper Quartile	Median Concentration	Lower Quartile	Upper Quartile		
PDE4DIP_2.32508	258.4734	121.7833	583.68	856.3743	494.0256	1501.917	0.301823	0.001
DOCK4_2.54606	24.13794	13.79644	49.05431	71.66497	31.79918	101.6345	0.336816	0.006
MAPK4_1.18194	403.9571	167.9237	1100.5	1191.75	661.2829	1904.942	0.338961	0.009
SFMBT2_2.16231	275.2411	158.8478	733.6667	792.375	471.9091	1289.031	0.347362	0.007
TRIM24_2.123	152.7522	102.8923	354.7179	437.3595	193.1333	639.5307	0.34926	0.009
NFIX_1.4822	548.7424	251.9655	1162.857	1528.05	631.4481	3530.375	0.359113	0.003
CACNA1B_1.21005	390.6557	251.3125	1100.5	1062.575	631.8065	1803.048	0.36765	0.0096
KLHL1_1.45240	742.1714	450.3871	1849	1935.205	1198.014	3826.25	0.383511	0.005
FAT3_2.34125	427.0625	319.875	864.625	1085.248	633.8409	1554.736	0.393516	0.007
PHACTR1_1.31134/circular	5.952787	3.91866	9.5	4.104908	3.541242	6.14427	1.450163	0.038

Abbreviations: a *p*-value less than 0.05 is considered as statistically significant.

**Table 2:** Calculated odds ratio for selected transcripts.

Target/Form	Odds Ratio GDM to Controls	95% Confidence Interval	*p*-Value
PTN_2.4369/linear	9.72	[1.148–82.318]	0.009
PHACTR1_2.43094/linear	8.31	[0.806–60.827]	0.018
KITLG_2.26281/linear	7.00	[0.970–71.141]	0.03

Abbreviations: a *p*-value less than 0.05 is considered as statistically significant.

**Table 3:** Spearman’s rank correlation between top 10 dysregulated transcripts and clinical variables.

lncRNA	Clinical Variables			
	CRP	INSULIN	FPG	HOMA-IR
PDE4DIP_2.32508	−0.04	−0.23	0.19	−0.17
DOCK4_2.54606	0.07	−0.08	0.18	−0.05
MAPK4_1.18194	0.05	−0.01	**0.3**	0.05
SFMBT2_2.16231	0.01	0.06	0.22	0.08
TRIM24_2.123	0.18	0.04	**0.35**	0.1
NFIX_1.4822	0.01	−0.04	**0.41**	0.03
CACNA1B_1.21005	−0.02	0.02	**0.37**	0.08
KLHL1_1.45240	−0.01	−0.12	**0.27**	−0.08
FAT3_2.34125	0.05	0.01	**0.34**	0.06
PHACTR1_1.31134	−0.12	−0.07	−0.2	−0.09

Abbreviations: CRP, C-reactive protein; FPG, fasting plasma glucose; HOMA-IR, Homeostatic Model Assessment of Insulin Resistance; lncRNA, long non-coding RNA. Significant correlations are in bold and marked in red.

**Table 4:** Correlation between selected targets and clinical variables.

Target/Form	Laboratory Parameter			
	CRP	Insulin	FPG	HOMA-IR
PTN_2.4369/linear	0.0003	0.002	0.11	0.02
PHACTR1_2.43094/linear	−0.05	−0.12	0.094	−0.08
KITLG_2.26281/linear	−0.097	−0.04	0.23	−0.002
Relative *PHACTR1* levels	−0.025	0.06	−0.17	0.02

Data are presented as Spearman’s rank correlation coefficients (rs). Abbreviations: CRP, c-reactive protein; FPG, fasting plasma glucose; HOMA-IR, Homeostatic Model Assessment of Insulin Resistance.

**Table 5:** Differences in expression of selected targets in patients with GDM divided by type of treatment.

Target/Form	GDM Treated by Insulin (n = 20)	GDM Treated by Diet (n = 14)	*p*-Value
PTN_2.4369/linear	11.77 [5.59–20.7]	4.2 [2.51–13.58]	0.06
PHACTR1_2.43094/linear	162.2 [42.16–318.66]	106.62 [52.28–165.49]	0.39
KITLG_2.26281/linear	1.75 [1.17–2.39]	0.76 [0.58–1.39]	0.02
Relative *PHACTR1* levels	0.025 [0.017–0.92]	0.069 [0.038–0.37]	0.07

Data are presented as median [lower–upper quartile]. Significant differences between groups are bolded.

**Table 6:** Potential interaction between *PHACTR1* (has_circ_0002268) and microRNAs.

Micro-RNA	Number of Sites	Context + Score Percentile
hsa-miR-1236	1	90
hsa-miR-1265	1	89
hsa-miR-1289	1	94
**hsa-miR-1299**	**1**	**96**
hsa-miR-1827	1	93
hsa-miR-31	1	93
hsa-miR-346	1	88
hsa-miR-370	1	78
hsa-miR-432	1	75
hsa-miR-516b	1	80
hsa-miR-556-5p	1	87
hsa-miR-558	1	76
hsa-miR-568	2	82 and 79
**hsa-miR-643**	**1**	**97**
**hsa-miR-661**	**1**	**99**
hsa-miR-766	1	85
hsa-miR-767-3p	1	NA
hsa-miR-924	1	92
**hsa-miR-942**	**1**	**96**

Abbreviation: hsa-miR, human microRNA. The top 4 microRNAs are bolded.

**Table 7:** Subjects’ characteristics.

	GDM (n = 34)	Controls (n = 28)	*p*-Value
Age [years]	33.1 ± 5.1	30.6 ± 3.8	0.04
BMI [kg/m^2^]	29.3 ± 4.4	27.3 ± 4.7	0.11
Primipara, n (%)	21 (61.8)	17 (60.7)	0.86
Birth by cesarean section, n (%)	23 (67.6)	13 (46.4)	0.15
Birth weight [g]	3450 [2820–3660]	2920 [2510–3520]	0.17
Week of delivery [weeks]	37 [36–38]	38 [37–40]	0.08
Insulin [mU/mL]	12.5 [8.8–17.2]	9.7 [7.4–16]	0.22
FPG [mg/dL]	77 [73–82]	78 [73–80.5]	0.72
HOMA-IR	2.5 [1.6–3.3]	1.9 [1.4–3]	0.24
CRP [mg/L]	4.4 [4–8]	4 [4–10.7]	0.81
Treatment of gestational diabetes with insulin, n (%)	20 (58.8)	None	not applicable
Treatment of gestational diabetes through diet, n (%)	14 (41.2)	None	not applicable

Data are presented as mean ± SD; number (%) or median [lower—upper quartile]. Abbreviations: BMI, body mass index; CRP, C-reactive protein; FPG, fasting plasma glucose; GDM, Gestational Diabetes Mellitus; HOMA-IR, Homeostatic Model Assessment of Insulin Resistance.

## Data Availability

Due to privacy and ethical concerns, the data that support the findings of this study are available on request from the last author (D.D.-K.).

## Supplementary Materials

The following supporting information can be downloaded at: https://www.mdpi.com/article/10.3390/ijms25137040/s1.
