# Correction: Dłuski et al. Circular RNA hsa_circ_0002268 (*PHACTR1*) Is Specific to Gestational Diabetes Mellitus in a Polish Pregnant Population. *Int. J. Mol. Sci.* 2024, *25,* 7040

**DOI:** 10.3390/ijms252413737

**Published:** 2024-12-23

**Authors:** Dominik Franciszek Dłuski, Marek Cieśla, Dorota Darmochwał-Kolarz

**Affiliations:** 1 Department of Obstetrics and Perinatology, Medical University of Lublin, 20-090 Lublin, Poland; 2 Institute of Medical Science, College of Medical Science, University of Rzeszow, 35-959 Rzeszow, Poland; mciesla@ur.edu.pl; 3 Department of Obstetrics and Gynecology, College of Medical Science, University of Rzeszow, 35-301 Rzeszow, Poland; ddarmochwal@ur.edu.pl

In the original publication [1], the acknowledgments for Poland—Clinical Scholars Research Training Program organized by Harvard Medical School and Medical Research Agency from Poland were not included. 

The authors state the following Acknowledgments: This article was prepared during Poland—Clinical Scholars Research Training Program organized by Harvard Medical School and financed by Medical Research Agency from Poland. The manuscript is authors’ own work and is not affiliated with Harvard or Harvard Medical School. 

The authors state that the scientific conclusions are unaffected. This correction was approved by the Academic Editor. The original publication has also been updated.
