# Supplementary material for: Circular RNA hsa_circ_0002268 (PHACTR1) Is Specific to Gestational Diabetes Mellitus in a Polish Pregnant Population
Source: Int J Mol Sci. 2024 Jun 27;25(13):7040. doi: 10.3390/ijms25137040 (PMC11241481; doi:10.3390/ijms25137040)
Supplement: Supplementary file 1 [file ijms-25-07040-s001.zip › ijms-3042593-supplementary.pdf]

Supplementary data

Table S1. Transcripts whose concentration was significantly different between patients with GDM and control group.

Target	GDM (n=34)			Controls (n=28)				
	Median	Lower quartile	Upper quartile	Median	Lower quartile	Upper quartile	RATIO [GDM : Controls]	p-value
**NA_PDE4DIP_2.32508**	258.47343	121.78333	583.68	856.37433	494.02564	1501.91667	0.301822953	**0.001**
**NA_DOCK4_2.54606**	24.137942	13.796443	49.054313	71.664971	31.799178	101.634456	0.336816461	**0.006**
**NA_MAPK4_1.18194**	403.95714	167.92366	1100.5	1191.75	661.28289	1904.94167	0.338961311	**0.009**
**NA_SFMBT2_2.16231**	275.24114	158.84783	733.66667	792.375	471.90909	1289.03125	0.347362225	**0.007**
**NA_TRIM24_2.123**	152.75219	102.89231	354.71795	437.35952	193.13326	639.530702	0.349260013	**0.009**
**NA_NFIX_1.4822**	548.74242	251.96552	1162.8571	1528.05	631.44805	3530.375	0.359112872	**0.003**
**NA_CACNA1B_1.21005**	390.65568	251.3125	1100.5	1062.575	631.80654	1803.04762	0.36764998	**0.0096**
**NA_KLHL1_1.45240**	742.17143	450.3871	1849	1935.2045	1198.0143	3826.25	0.383510586	**0.005**
**NA_FAT3_2.34125**	427.0625	319.875	864.625	1085.2481	633.84091	1554.73571	0.393516001	**0.007**
**NA_PDE4DIP_3.60116**	0.1917015	0.1273504	0.3781323	0.4809926	0.2774492	0.76549506	0.398553887	**0.02**
**NA_NTRK2_1.3401**	717.41304	399	1621.3333	1770.6161	1162.6957	2969.75	0.405177077	**0.001**
**NA_SMO_2.818**	4.65382	2.5190993	8.8793325	11.402127	5.9937656	17.4942733	0.408153667	**0.019**
**NA_GLIS3_1.72171**	229.29658	113.96078	389.70968	553.40861	321.76742	970.74386	0.414335047	**0.005**
**NA_MAPK4_2.39**	2.2351157	1.300602	5.550873	5.1531505	2.7218478	8.42566616	0.433737703	**0.037**
**NA_BMPR2_2.16087**	1.0794578	0.6178725	2.040413	2.4249301	1.2285905	3.37139213	0.445150061	**0.031**
**NA_CTC-525D6.1_1.6930**	222.77273	132.2	512.22222	497.78383	324.84531	901.757692	0.447529051	**0.006**
**NA_SH3GL3_2.7817**	15.945755	8.7021277	31.106977	34.349685	22.529514	79.9559874	0.464218383	**0.005**
**NA_NONO_1.12874**	196.66162	88.07619	335.05455	417.52127	239.82276	570.478333	0.471021795	**0.006**
**NA_PDE4DIP_1.10830**	282.39216	173.94737	464.65385	594.65625	344.9375	792.081454	0.474883022	**0.001**
**NA_ARPP21_2.46207**	351.05793	194.41176	1024.4444	726.5119	467.7	1136.49231	0.483210159	**0.036**
**NA_CTC-525D6.1_2.26133**	1.3585405	0.5789557	2.5171741	2.7714111	1.1228606	4.1089126	0.49019811	**0.031**
**NA_E2F4_1.359**	313.88258	169.92632	576.25	636.52941	402.12007	859.497778	0.493115589	**0.016**
**NA_LDB2_2.20291**	1.2730092	0.5910229	2.7755359	2.563709	1.6354516	4.20930886	0.496549795	**0.034**
**NA_IFT46_2.4270**	2.3602903	1.3145195	4.5762488	4.7529736	2.4018241	7.33026209	0.496592348	**0.033**
**NA_MYT1L_2.758**	1100.8542	451.58824	3137.5	2216.125	1532.125	5630.66667	0.496747325	**0.025**
**NA_ZNF562_2.49612**	1.8907638	1.0116295	3.9156881	3.7899038	2.1577869	5.83541351	0.498894928	**0.036**
**NA_HECTD1_1.21843**	298.27141	176.63636	691.7	588.05455	343.07589	1014.8254	0.507217244	**0.046**
**NA_SEZ6L2_1.13503**	813.55263	458.29167	2194.6667	1592.4118	1013.3043	3483.25	0.510893382	**0.034**
**NA_FAT3_1.20736**	411.88603	165.09677	733.66667	800.39286	408.80391	1200.66667	0.514604829	**0.029**
**NA_LPHN3_2.51920**	1.6240603	1.0692004	3.5442834	3.1432137	1.9179283	6.37204058	0.516687835	**0.022**
**NA_LRRC7_1.2825**	786.61905	368.52941	1459.2	1522.2	820.1	2445.11111	0.516764583	**0.026**
**NA_UNC79_2.41215**	861.36364	522.08333	1667.3	1650.5	916.6875	2962.8	0.521880422	**0.021**
**NA_RBM39_2.1131**	2.5552007	1.3547844	4.4262078	4.8753629	2.6172412	7.90137296	0.524104711	**0.035**
**NA_KCNN2_2.8231**	1.5518829	0.7159484	3.0408971	2.954377	1.5736745	6.28546823	0.525282625	**0.027**
**NA_NRXN1_1.63784**	670.65	272.3913	1417.5385	1275.4167	812.15179	2170.375	0.525828161	**0.043**

**NA_SCMH1_2.7684**	1.2945977	0.6861033	2.4080012	2.4476329	1.3434273	3.81263336	0.528918237	**0.033**
**NA_NRXN1_2.3460**	2.1862891	1.1849367	4.6829787	4.1178887	2.3747807	7.57038095	0.530924768	**0.027**
**NA_KLHL1_2.13097**	3.670498	1.79754	8.4204717	6.8514136	4.0359616	14.2726696	0.535728569	**0.018**
**NA_SLC2A13_2.1083**	1.8736819	1.0268808	3.9109765	3.4677598	2.0103124	5.93379937	0.540314802	**0.034**
**NA_ZKSCAN1_2.6350**	0.8194752	0.4014739	1.7339901	1.5136119	1.1015132	3.01100403	0.541403767	**0.022**
**NA_GLIS2_2.27892**	138.72235	36.571429	338.98333	255.77	110.32615	817.28125	0.542371449	**0.034**
**NA_LRRC7_2.21457**	1.3744537	0.6704801	2.7679367	2.4569594	1.2398069	3.85378711	0.559412454	**0.043**
**NA_PTN_2.4369**	9.9032977	3.0317889	18.632963	17.621626	8.3031254	26.5972175	0.561996803	**0.036**
**NA_ZNF609_2.36168**	2.494478	1.3696579	4.4826884	4.4305994	2.2866278	7.72277493	0.563011423	**0.042**
**NA_FOXN3_2.35150**	1.0706339	0.6713524	1.8909446	1.8858698	1.1324778	2.50912181	0.567713609	**0.043**
**NA_DPYSL5_1.22402**	668.58134	337.91304	1536.6667	1133.994	829.77679	2156.5	0.589581046	**0.036**
**NA_EPHB2_1.47378**	433.01122	231.55789	703.28261	726.5	458.34375	1035.05714	0.596023699	**0.038**
**NA_TMEM165_2.1663**	2.3738143	1.3271795	4.1288359	3.9771249	2.5993521	6.869807	0.596866915	**0.033**
**NA_PHACTR1_2.43094**	126.82189	50.604839	227.3662	207.32381	81.694987	904.940217	0.61170923	**0.022**
**NA_ZNF398_2.9865**	14.02928	9.6880513	21.364829	22.826665	12.94095	47.4338281	0.614600502	**0.029**
**NA_YY1AP1_2.13236**	1.4059971	0.6405276	2.3787385	2.0511276	1.4406911	4.02183079	0.685475193	**0.048**
**NA_SLAIN2_2.72953**	1.2789275	0.7669202	1.726717	1.7147823	1.3974991	2.34580292	0.745825003	**0.043**
**NA_KITLG_2.26281**	1.4496957	0.7556599	2.3015477	1.9300648	1.3854092	2.92572039	0.751112441	**0.024**
**N_HERC4_1.15252**	0.000309	0.000278	0.0003963	0.0003973	0.0003299	0.00047965	0.777746973	**0.018**
**NA_KLHL8_2.46355**	2.405197	1.641959	3.4490152	3.0652831	2.4693786	4.33779884	0.784657374	**0.048**
**N_HERC1_1.40264**	0.0012995	0.0011308	0.0016082	0.0015345	0.001284	0.00199627	0.846885218	**0.048**
**NA_PHACTR1_1.31134**	5.9527866	3.9186603	9.5	4.1049078	3.5412419	6.1442697	1.450163282	**0.038**

Abbreviations: GDM; gestational diabetes mellitus. Data were calculated by Mann-Whitney U test and two-sided p-values were presented.

Table S2. Spearman rank correlation between transcripts and laboratory parameters.

**Target**	**Laboratory parameters**			
	CRP	Insulin	FPG	HOMA-IR
NA_ARPP21_2.46207	0.004481	0.027575	0.315243	0.071391
NA_BMPR2_2.16087	0.076757	0.030722	0.326995	0.082521
NA_CACNA1B_1.21005	-0.018609	0.020020	0.369667	0.078190
NA_CTC-525D6.1_1.6930	-0.037545	-0.036061	0.304071	0.009645
NA_CTC-525D6.1_2.26133	0.084627	-0.036288	0.245436	0.006119
NA_DOCK4_2.54606	0.068997	-0.084713	0.175855	-0.048123
NA_DPYSL5_1.22402	-0.010411	-0.049458	0.305962	0.003148
NA_E2F4_1.359	0.211746	0.117954	0.331535	0.162952
NA_EPHB2_1.47378	0.024153	0.012665	0.317249	0.063141
NA_FAT3_1.20736	-0.081075	0.040896	0.172677	0.075269
NA_FAT3_2.34125	0.048066	0.007580	0.338495	0.059908
NA_FOXN3_2.35150	0.137475	0.028103	0.285938	0.073556
NA_GLIS2_2.27892	-0.102389	0.048451	0.219132	0.096573
NA_GLIS3_1.72171	0.061564	-0.040518	0.330400	0.006623
NA_HECTD1_1.21843	0.027899	0.055879	0.277162	0.093551

N_HERC1_1.40264	0.030714	-0.123267	-0.121734	-0.133439
N_HERC4_1.15252	0.009946	-0.016872	0.008121	-0.028128
NA_IFT46_2.4270	0.059706	-0.008134	0.319026	0.042986
NA_KCNN2_2.8231	0.039567	0.018736	0.312973	0.069427
NA_KITLG_2.26281	-0.097552	-0.036993	0.230960	-0.002241
NA_KLHL1_1.45240	-0.014182	-0.121506	0.274845	-0.081918
NA_KLHL1_2.13097	0.042682	0.015865	0.328761	0.073808
NA_KLHL8_2.46355	0.085064	0.051598	0.300313	0.095717
NA_LDB2_2.20291	0.092524	0.041677	0.296328	0.089220
NA_LPHN3_2.51920	0.051372	-0.015915	0.287779	0.029790
NA_LRRC7_1.2825	0.092989	0.034298	0.401519	0.093249
NA_LRRC7_2.21457	0.020221	0.010098	0.270579	0.057692
NA_MAPK4_1.18194	0.050471	-0.011030	0.299510	0.046285
NA_MAPK4_2.39	0.036316	-0.014530	0.358066	0.040618
NA_MYT1L_2.758	0.026946	0.028208	0.243663	0.051629
NA_NFIX_1.4822	0.013007	-0.042029	0.406689	0.033719
NA_NONO_1.12874	-0.067931	-0.170257	0.168239	-0.122158
NA_NRXN1_1.63784	0.080528	0.062981	0.324852	0.111329
NA_NRXN1_2.3460	0.023773	-0.004785	0.296908	0.044799
NA_NTRK2_1.3401	0.039431	-0.054042	0.278603	-0.010929
NA_PDE4DIP_2.32508	-0.041617	-0.225155	0.185918	-0.166730
NA_PDE4DIP_3.60116	0.013198	-0.018862	0.320489	0.031503
NA_PDE4DIP_1.10830	0.028145	0.083001	0.226496	0.110725
NA_PHACTR1_1.31134	-0.116106	-0.070258	-0.200671	-0.088867
NA_PHACTR1_2.43094	-0.048339	-0.119490	0.094068	-0.078593
NA_PTN_2.4369	0.000301	0.001687	0.110839	0.015285
NA_RBM39_2.1131	0.059351	-0.018308	0.298321	0.028027
NA_SCMH1_2.7684	-0.015767	0.008084	0.309871	0.054670
NA_SEZ6L2_1.13503	0.052222	-0.074417	0.147213	-0.038908
NA_SFMBT2_2.16231	0.009701	0.057365	0.215374	0.076528
NA_SH3GL3_2.7817	0.002733	-0.012793	0.368279	0.045957
NA_SLAIN2_2.72953	0.176851	0.060916	0.314612	0.102012
NA_SLC2A13_2.1083	0.073314	-0.000327	0.333981	0.052655
NA_SMO_2.818	0.092961	0.002518	0.331106	0.059354
NA_TMEM165_2.1663	-0.004345	-0.040115	0.244603	-0.007479
NA_TRIM24_2.123	0.177671	0.044623	0.348482	0.098889
NA_UNC79_2.41215	0.062568	0.037335	0.342708	0.097144
NA_YY1AP1_2.13236	0.066538	0.015689	0.343060	0.068822
NA_ZKSCAN1_2.6350	0.029348	0.009594	0.362454	0.066606
NA_ZNF398_2.9865	0.117882	0.069377	0.319581	0.119942
NA_ZNF562_2.49612	0.035332	-0.021355	0.297942	0.025862
NA_ZNF609_2.36168	0.022434	-0.015009	0.238223	0.023444

Data are presented by Spearman's rank correlation coefficients (r_s). Significant correlations are marked by red. Abbreviations: CRP, c-reactive protein; FPG, fasting plasma glucose , HOMA-IR, Homeostatic Model Assessment of Insulin Resistance.
